# Medical Banquets

**Published:** 1917-04

**Authors:** 


### Medical Banquets.

Occasionally there come rumors that some of the banquets given by associations of medical men are just a bit risque and that the after-dinner speeches are not of a kind that can be reported in full in the newspapers. In fact some of those attended by "men only" are said to border rather near the indecent, and even where ladies attend they have occasion to blush at some of the stories and to wish they were elsewhere.

Just why wine and vulgarity should be regarded as essential ingredients of a menu for physicians is hard to understand, for doctors should be the gentlest of gentlemen. The nature of the profession is such that a vulgar minded man should be ostracised by all who respect the profession, and vulgar speech even to "men only" is a sure evidence of a low order of moral thought. The profession of medicine is almost sacred in its character, and if it is not so regarded by its members they have a wrong perception of their calling. Men and women are under the necessity of confiding to their physicians the most delicate, the most intimate, the most sacred affairs of life, and self-respecting persons do not like to commit their secrets of this kind to a mental libertine. They will not do so if they know it.

The physician who indulges in indecent stories, even if women are not present, isn't decent enough to go in a professional way into respectable families. Physicians may not be worse offenders as a class than others, but their profession demands that they should not descend to improprieties of speech or thought. Purity of mind and conduct is evidenced by purity of speech. Men are judged by their speech.

While no kind of vulgarity on the part of a physician is tolerable, the most inexcusable of all disgusting stories are those based on the professional relations of physician to patient, in which the physician undertakes to place an obscene construction on professional experiences. It it said that some physicians are given to this in their after-dinner talks, using what should be the most sacred confidences as a basis for insinuating suggestions. This practice, happily, is not common, and most physicians frown on it, but frowning is not a rebuke to the evil-minded man.

The fellow who has such a low conception of his position as to resort to vulgar stories when called upon to talk at a dinner need not be surprised to find himself finally left off the list of speakers entirely, and the physician who narrates his professional experiences as a basis for a display of his own low-mindedness may expect his fellow practitioners to lose all the respect they have ever had for him.

Physicians should be clean-mouthed, both in their public utterances and in their private conversations, if they would expect to sustain reputations of a kind that will entitle them to enter the homes of people of refinement.

